# Outcomes and Toxicities of Modern Combined Modality Therapy with Atezolizumab Plus Bevacizumab and Radiation Therapy for Hepatocellular Carcinoma

**DOI:** 10.3390/cancers14081901

**Published:** 2022-04-09

**Authors:** Gohar Shahwar Manzar, Brian Sandeep De, Chike Osita Abana, Sunyoung S. Lee, Milind Javle, Ahmed O. Kaseb, Jean-Nicolas Vauthey, Hop Sanderson Tran Cao, Albert C. Koong, Grace Li Smith, Cullen M. Taniguchi, Emma Brey Holliday, Prajnan Das, Eugene Jon Koay, Ethan Bernard Ludmir

**Affiliations:** 1 Department of Radiation Oncology, The University of Texas MD Anderson Cancer Center, Houston, TX 77030, USA; gsmanzar@mdanderson.org (G.S.M.); bsde@mdanderson.org (B.S.D.); coabana@mdanderson.org (C.O.A.); 2 Department of Gastrointestinal Medical Oncology, The University of Texas MD Anderson Cancer Center, Houston, TX 77030, USA; sslee1@mdanderson.org (S.S.L.); mjavle@mdanderson.org (M.J.); akaseb@mdanderson.org (A.O.K.); 3 Department of Surgical Oncology, Division of Surgery, The University of Texas MD Anderson Cancer Center, Houston, TX 77030, USA; jvauthey@mdanderson.org (J.-N.V.); hstran@mdanderson.org (H.S.T.C.); 4 Department of Gastrointestinal Radiation Oncology, The University of Texas MD Anderson Cancer Center, Houston, TX 77030, USA; ackoong@mdanderson.org (A.C.K.); glsmith@mdanderson.org (G.L.S.); ctaniguchi@mdanderson.org (C.M.T.); ebholliday@mdanderson.org (E.B.H.); prajdas@mdanderson.org (P.D.); 5 Department of Biostatistics, The University of Texas MD Anderson Cancer Center, Houston, TX 77030, USA

**Keywords:** hepatocellular carcinoma, atezolizumab, bevacizumab, radiation therapy, RT, immunotherapy

## Abstract

**Simple Summary:**

Hepatocellular carcinoma (HCC) is a leading cause of cancer-related death worldwide with slow progress in the development of effective therapies. The breakthrough IMbrave150 trial established atezolizumab plus bevacizumab as the standard of care first-line therapy for patients with unresectable/advanced HCC, demonstrating improved overall survival. Radiation therapy (RT) is a locoregional treatment that may prevent morbidity from tumor-related vascular compromise and associated liver failure. There is no documented evidence yet describing the safety and outcomes of combination RT and atezolizumab/bevacizumab. In the retrospective review of our experience, we identified 21 patients with advanced HCC who underwent liver-directed RT with atezolizumab/bevacizumab. From our findings, the treatment is well-tolerated, safe, and does not potentiate liver failure. There were uncommon autoimmune or GI bleeding events in some patients, mostly unrelated to RT. Post-RT absolute lymphocyte counts (ALC) improved more quickly with atezolizumab/bevacizumab than they did without, which may be a favorable treatment prognosticator.

**Abstract:**

Atezolizumab plus bevacizumab has become frontline therapy for unresectable HCC. The compatibility of atezolizumab/bevacizumab with liver-directed RT has not been reported. Methods: HCC patients treated with liver-directed RT and atezolizumab/bevacizumab between 1/2020–11/2021 were included. Toxicity and outcomes were retrospectively recorded. For ALCs, we matched the analysis to a previously cohort of RT-treated HCC patients who did not receive atezolizumab/bevacizumab. Survival and time-to-liver-failure were analyzed using Kaplan–Meier. Results: Of 21 patients, with a median follow-up of 9.5 months, the median OS was 16.1 months. Post-RT, all patients had reduced tumors or treatment response. There were no ≥Grade 3 RT-related toxicities. Autoimmune complications occurred in two patients (9.5%), and GI bleeding in three patients (14.3%). Liver function remained stable post-RT. There was a marked decrease in ALCs immediately post-RT (post-RT/pre-RT ratio 47.3%, *p* < 0.0001), restored by 1 month to pre-treatment baseline (1-month post-RT/pre-RT ratio 95.1%, n.s.). Compared to HCC patients treated with RT alone, post-RT ALC recovery was faster with atezolizumab/bevacizumab (*p* = 0.009). Conclusion: In this first reported experience of RT with modern systemic therapy for HCC, combination therapy is safe and well-tolerated. As a favorable prognosticator, there appears to be faster recovery of ALC among patients who received RT with atezolizumab/bevacizumab.

## 1. Introduction

Hepatocellular carcinoma (HCC) is the seventh most common malignancy and fourth most common cause of cancer-related death worldwide, with a disproportionately high socioeconomic burden on low-resource countries and vulnerable populations [1,2]. Patients with early-stage disease may be treated definitively with resection, radiofrequency ablation, trans-arterial chemoembolization, or liver transplantation [3], but often recur with HCC elsewhere unless they undergo liver transplantation. Most patients are diagnosed with unresectable HCC, which lends a poor prognosis if coupled with poor liver function. Until recently, there was slow improvement in the realm of systemic treatments for these patients.

In 2008, the SHARP trial demonstrated modestly longer survival for untreated advanced HCC with the multikinase small molecule inhibitor sorafenib [4]. For over a decade, several clinical trials with other therapeutic approaches failed, and there was an inability to replace sorafenib as first-line therapy despite poor outcomes [5]. In 2018, the REFLECT trial revealed noninferior survival with lenvatinib, which became an alternative treatment option to sorafenib [6]. A breakthrough finding emerged in 2020 from the IMbrave150 trial, which established the frontline indication for the anti-PD-L1 inhibitor atezolizumab in combination with the anti-angiogenic monoclonal antibody bevacizumab [7]. Compared to sorafenib, atezolizumab plus bevacizumab offered superior overall survival as first-line therapy for patients with unresectable/advanced HCC. This has led to a dramatic departure away from targeted therapy in favor of this doublet combination as the new standard of care for patients with unresectable disease [8]. This is particularly true for patients with Child-Pugh A disease so as to avoid bevacizumab-related variceal bleeding, which may pose a higher risk in patients with more advanced cirrhosis [9]. Bolstered by the results of the IMbrave150 trial, there has been substantial interest devoted to role of immune-checkpoint inhibitors (ICI) in the treatment of HCC [10,11].

Radiation therapy (RT) is a locoregional therapy that may be utilized to safely consolidate liver lesions to prevent morbidity from tumor-related vascular compromise and associated tumor-related liver failure, particularly through more conformal technology, such as intensity-modulated radiation therapy (IMRT) or proton radiation therapy [12]. While RT has been given to patients undergoing systemic therapy with atezolizumab plus bevacizumab since the transition of standard of care, there is no documented evidence on the safety and outcomes of combination RT and atezolizumab/bevacizumab.

Here we report our experience using RT with atezolizumab plus bevacizumab to assess the safety and efficacy of this modern combined modality approach.

## 2. Materials and Methods

### 2.1. Patient Selection

**Figure 1:**
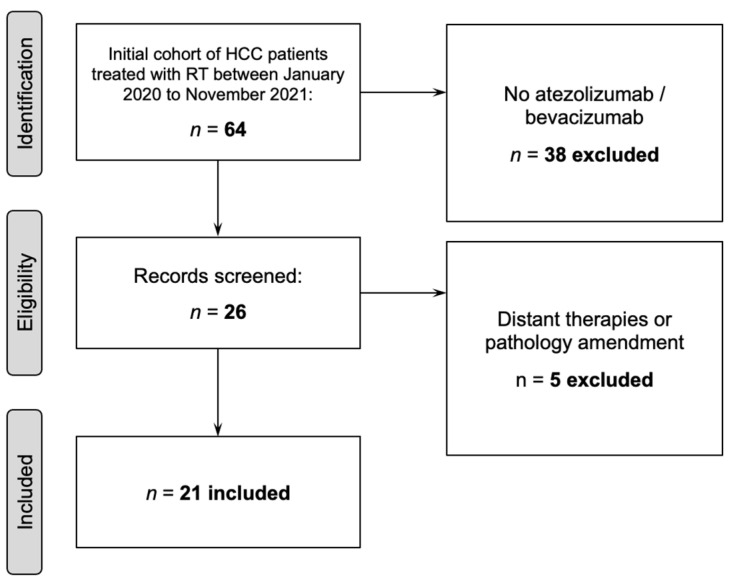
Patient inclusion and exclusion criteria. *Abbreviations:* RT = radiation therapy.

We identified patients with HCC age ≥18 treated with definitive RT with atezolizumab plus bevacizumab between 1/2020–11/2021 at the University of Texas MD Anderson Cancer Center. Previous therapies were allowed. The retrospective review of these medical records was approved by the Institutional Review Board. Exclusion criteria consisted of not having received atezolizumab (Roche, Basel, Switzerland), or bevacizumab (Roche, Basel, Switzerland), distant timing of therapies (≥2 months apart), or pathology amendment (Figure 1). Patients were staged using the AJCC 8th edition.

Twenty-one patients met inclusion criteria (Figure 1).

### 2.2. Treatment

Patients typically underwent multidisciplinary evaluation with a recommendation for combined modality therapy with atezolizumab and bevacizumab to be given neoadjuvantly for a period or initiated at the time of RT. RT was recommended for well-selected patients with HCC, including those with portal venous tumor thrombosis (PVTT), with the goal of improving local control, reducing morbidity, and potentially prolonging the freedom from venous obstruction and liver failure. Bevacizumab was held during RT to reduce the risk of gastrointestinal bleeding. Treatment with a passive-scatter proton beam therapy, IMRT, or 3D conformal radiation was determined at the discretion of the physician and ultimately by the medical policy of the patient’s insurance carrier. Techniques, treatment processes, dosimetric, planning, quality assurance, and follow-up considerations are summarized in our prior literature [13].

CT simulation with contrast was performed in the supine position with immobilization and motion management considerations, including either deep inspiration breath hold technique or four-dimensional simulation with capture of fields of interest across the complete respiratory cycle. The gross primary tumor volume (GTV) and clinical tumor volume (CTV) were defined by cross-sectional imaging, with a volumetric expansion to generate the planning target volumes (PTV). All RT plans conformed to strict dose constraints to luminal structures as per AAPM TG101 for ≤5 fractions [14], and our prior published work for 10–15 fractions [15]. For regimens ≤15 fractions, we always implemented planning risk volume (PRV-GI) created by a 5 mm expansion of adjacent organs at risk, typically including the stomach, small bowel, duodenum, large bowel, esophagus, and heart. All contours and plans were validated in a departmental quality assurance chart rounds to ensure appropriate target coverage and safe avoidance of organs at risk. Patients were treated with a simultaneous integrated boost (SIB) approach [16]. Different dose levels were considered based on functional status, definitive vs. palliative intent, and proximity to organs at risk, particularly luminal structures. When feasible, dose-escalation to an ablative biologically equivalent dose (BED) of ≥75 Gy was preferred, with priority given to safely meeting normal organ dose constraints [13]. Patients were evaluated weekly during treatment.

Raystation (RaySearch Laboratories, Stockholm, Sweden) was used for 3D and IMRT planning to generate volumetric full or partial arcs. Eclipse (Varian, Palo Alto, CA) software was used for proton therapy planning. All patients were monitored daily for set up accuracy using either on-board kilovoltage cone-beam CT (IMRT) or orthogonal X-rays (proton therapy). Proton therapy patients underwent weekly verification CTs in the treatment position.

Standard doses were delivered for atezolizumab (1200 mg) and bevacizumab (15 mg/kg of body weight) in 3-week cycles unless the treating medical oncologist determined any indication for dose adjustment of systemic therapy. The timing of systemic therapy was heterogenous in the cohort. Not all patients received neoadjuvant or adjuvant systemic therapy, but where it was utilized, its initiation timepoint relative to the start of RT was noted. If systemic therapy was not given concurrently, inclusion of these patients necessitated that they received systemic therapy doses within 2 months of RT initiation (neoadjuvant) or completion (adjuvant).

### 2.3. Data Collection

Demographic data, disease features, treatment characteristics, toxicity, and clinical/radiographic outcomes were recorded. Provider-assessed toxicities from on-treatment weekly management visits were graded according to Common Terminology Criteria for Adverse Events (CTCAE) v4.03. Child–Pugh scores (CPS) at diagnosis, prior to RT, and at the peak were calculated to assess liver decompensation, defined as an increase by ≥2 points. Radiographic reports documenting decreased tumor size, treatment response, or radiation-related changes were determined by interpreting radiologists. Lab values, including liver transaminases, bilirubin levels, and serial absolute lymphocyte counts (ALC), were collected pre-neoadjuvant therapy (pre-NAT), where applicable, then pre-RT, post-RT, and then 1 month, 2 months, 3 months, and 4 months post-RT. Albumin-Bilirubin (ALBI) grade for HCC was calculated for each patient at each timepoint. For ALC counts, we matched the analysis to a previously published cohort of RT-treated HCC patients who did not receive atezolizumab or bevacizumab [17]. The change in ALC per patient was compared to the pre-treatment baseline (∆) with ratios calculated for each patient at each timepoint. This ratio was then compared between the two groups of patients who received RT either with or without atezolizumab or bevacizumab for HCC. The final designations regarding suspected cause of death were based on the review and consensus of three independent physicians, with attributions assigned on a scale of “not related”, “unlikely related”, “possibly related”, and “definitely related”.

### 2.4. Statistical Analysis

Baseline patient characteristics were compared using the Mann-Whitney-Wilcoxon test for continuous variables and χ^2^ test for discrete variables. Tests of normality were performed for ALC with the Shapiro-Wilk test, and no normality violations were identified across the timepoints. Overall survival (OS) was calculated both from the date of diagnosis and from the start of RT, analyzed using the reverse Kaplan-Meier method. Distant and regional progression-free survival were computed from the end of RT and defined as lack of radiographic progression in the areas outside the irradiated region. Univariate analyses were conducted using Cox proportional hazards regression modeling; *p* < 0.05 was considered statistically significant. Statistical analyses and graphs were generated using GraphPad Prism v9.0 (La Jolla, CA, USA) or Stata Version 13.0 (StataCorp, College Station, TX, USA).

## 3. Results

### 3.1. Patient, Tumor, and Treatment Characteristics

Demographic and clinical data are presented in Table 1. The median age was 68 (range 38–78) years. Eighteen patients (85.7%) were graded as having ECOG 0–1 performance status at the time of RT; the other three patients (14.3%) had an ECOG of 2. The majority of the cohort was male (81%). Approximately two-thirds of the patients had pre-RT Child–Pugh scores (CPS) of 5–6 A (66.6%), with viral etiology of their cirrhosis, and had localized T4 disease. Seventeen (81.9%) of the patients had BCLC C disease. Fifteen (71.4%) of the patients had the high-risk feature of portal vein tumor thrombosis (PVTT).

The mean dose of radiation to the functional liver was 17.4 Gy (median in the cohort), with a maximum in the cohort receiving 21.9 Gy. This was below our mean functional liver dose constraint of 24 Gy for CPS A patients and approximated the 18 Gy constraint we apply for CPS B patients. We did not exceed the volumetric constraint requiring that 700 cc^3^ of normal liver receive ≤ 20 Gy. Two-thirds of the patients received IMRT and one-third underwent proton therapy, most commonly to a dose of 60–67.5 Gy (range 20–75 Gy) in 15 fractions (range 5–25).

One-third of the patients received prior systemic therapies, such as lenvatinib (Eisai, Inc. and Merck, Kenilworth, NJ, USA), nivolumab (Bristol-Myers Squibb, New York, NY, USA), sorafenib (Bayer and Onyx Pharmaceuticals, San Francisco, CA, USA), ramucirumab (Eli Lilly and Company, Indianapolis, IN, USA), or regorafenib (Bayer HealthCare, Leverkusen, Germany), some in combination with local therapies (9.5%) such as resection, TACE, or ablation, detailed in Table 2. RT was recommended for 15 patients (71.4%) due to the presence of PVTT, either as first-line therapy in seven of those patients or at the time of disease progression with presence of tumor thrombus. For the other six patients without PVTT (28.6%), liver-directed RT was indicated for the growth of disease occupying the central liver, or in the event of marked progression of a liver lesion in the context of other stable or decreasing disease.

**Figure 2:**
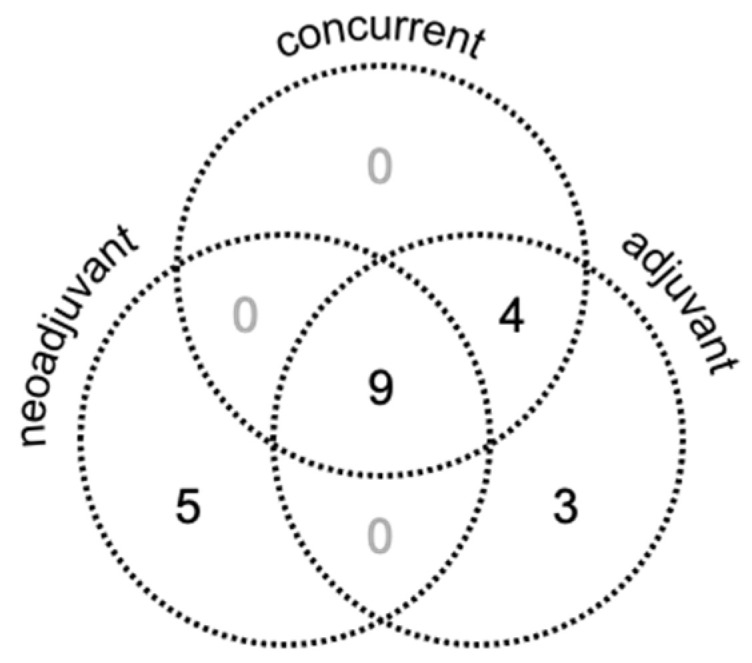
Timing of systemic therapy with RT. Patients in this cohort received a combination of neoadjuvant or adjuvant atezolizumab plus bevacizumab, and/or concurrent atezolizumab only.

The cohort was heterogenous with respect to systemic therapy sequencing with RT (Figure 2). Of the 21 patients, 14 (66.7%) received neoadjuvant atezolizumab plus bevacizumab. Thirteen patients (61.9%) received concurrent atezolizumab; no patient received concurrent bevacizumab. Sixteen patients (76.2%) received adjuvant atezolizumab plus bevacizumab. Three patients (14.3%) received only adjuvant atezolizumab plus bevacizumab.

### 3.2. Overview of Clinical Outcomes

With a median follow up of 9.5 months (95% confidence interval [CI]: 1.7–undefined) from the start of RT and 25.7 months (95% CI: 7.4–undefined) from diagnosis, 9 of the 21 patients (42.9%) were alive at last follow-up, and 12 (57.1%) had expired.

**Figure 3:**
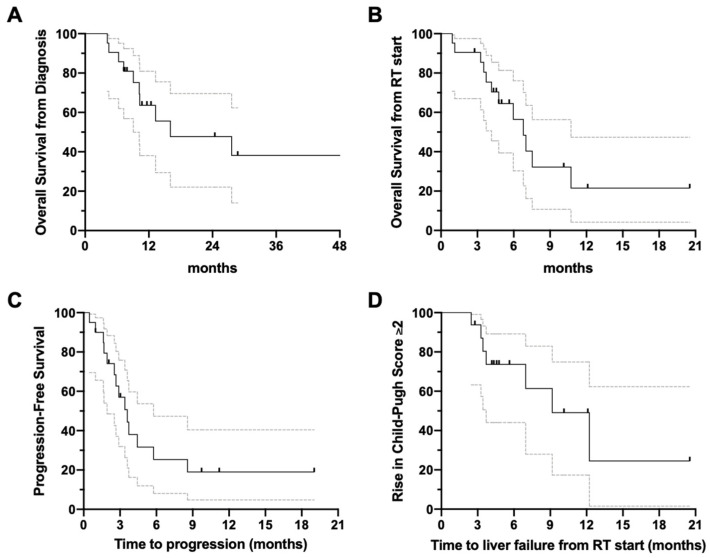
Time-to-event outcomes of the single-arm cohort. Graphs with 95% confidence intervals depict the (**A**) overall survival from diagnosis, (**B**) overall survival from the start of RT, (**C**) distant and regional progression-free survival following the completion of RT, and (**D**) time-to-liver decompensation from the start of RT, defined as an increase of ≥2 points. Abbreviations: RT = radiation therapy.

Among all patients, the median overall survival (OS) was 16.1 months from the date of diagnosis; the OS at 12 months was 61% (95% CI: 32–81%) (Figure 3A). From the start of RT, the median OS was 6.8 months (Figure 3B). The median distant and regional progression-free survival (Figure 3C) following the start of RT was 3.6 months at the time of last follow-up. A worsening of CPS by ≥2 developed in 12 patients (57.1%) from the start of RT due to tumor progression, with a median of 9.1 months to liver failure (Figure 3D). The estimated freedom from liver decompensation 12 months following the start of RT was 36% (7–67%). No factors on univariate analysis were associated with worse or improved survival or freedom from liver decompensation.

Of 20 radiographically evaluable patients, with a median radiographic follow-up of 3 months (range: 2 weeks to 19 months) from the completion of RT to a median GTV of 149.4 cc^3^ (range: 25.1–3426.9), 17 (85%) had reduced size of the irradiated lesions. Three patients (15%) demonstrated a stable lesion with imaging characteristics of treatment response from RT.

### 3.3. Acute Toxicity

**Figure 4:**
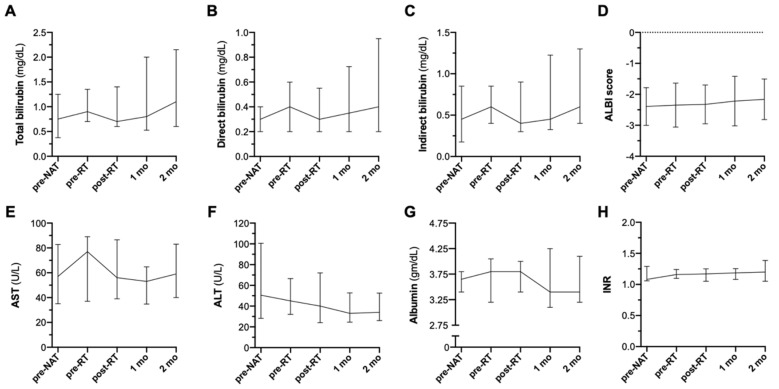
RT with atezolizumab plus bevacizumab does not potentiate acute liver decompensation. Stable transaminase levels were seen 2 months post-treatment as seen in the levels of total (**A**), direct (**B**), and indirect (**C**) bilirubin, ALBI scores (**D**), as well as AST (**E**), ALT (**F**), albumin (**G**), and INR (**H**) depicted with medians and interquartile ranges (IQR). Comparisons across all time points were statistically insignificant as measured by Wilcoxon signed-rank tests for continuous variables. Abbreviations: mo = month.

Objectively, the delivery of RT in all sequences with atezolizumab plus bevacizumab did not appear to potentiate acute liver hepatitis or decompensation, as demonstrated by stable levels of bilirubin, AST, ALT, albumin, INR, and ALBI (Albumin-Bilirubin) scores seen up to 2 months post-RT (Figure 4A–F).

During RT weekly management visits or at the end of treatment, there were no ≥Grade 3 toxicities (Table 3). The most common toxicities included Grade 1 fatigue (57.1%), nausea (47.6%), or abdominal pain (33.3%).

Three patients (14.3%) had gastrointestinal bleeding, two of whom had Child-Pugh A cirrhosis, not related to the direct impact of RT both temporally and by low radiation dose to the site of bleeding. Of these three patients, one incurred a bleeding duodenal ulcer 6 months after the completion of proton RT, with receipt of two neoadjuvant courses of atezolizumab plus bevacizumab. Following RT, he was treated with lenvatinib and then a STAT3 inhibitor. For the Grade 4 duodenal ulcer (catastrophic bleeding requiring urgent intervention), he underwent endoscopic placement of an OTSC clip, which halted the hemorrhage, and the patient was ultimately discharged. The maximum RT dose to the duodenum given 6 months prior to the development of the duodenal ulcer was 41.2 Gy–this was below the duodenal constraint of 45 Gy in this plan delivering 60 Gy in 15 fractions to the target via 4 D CT respiratory motion tracking. Two weeks later, this patient returned with abdominal hematomas from liver tumor hemorrhage. While in the intensive care unit (ICU) for hemorrhagic shock from the Grade 5 hemorrhage from the liver tumor (catastrophic bleeding causing death), the patient developed abdominal compartmental syndrome, acute renal failure requiring continuous renal replacement therapy, and multiorgan failure, leading to demise.

The second patient developed Grade 3 melena (invasive intervention; hospitalization) after three neoadjuvant cycles of atezolizumab plus bevacizumab, prior to the first day of RT. This patient was admitted to the hospital as well as hepatic encephalopathy. He underwent EGD, which disclosed multiple Grade 3, large (>5 mm) esophageal varices that were banded. He was started on a pantoprazole drip and lactulose with resolution of bleeding. He eventually discharged in stable condition but remained somnolent with progression of hepatic encephalopathy noted 2 weeks after finishing RT. The maximum dose to the esophagus was 26.9 Gy, given after he had already melena. He eventually succumbed to his disease 1 month after RT completion.

The third patient had a history of asymptomatic Grade 2 esophageal varices discovered at diagnosis that were banded prior to any receipt of therapy. He began only atezolizumab concurrently with RT. Following RT completion (with a point maximum dose to the esophagus of 3.5 Gy), he began atezolizumab plus bevacizumab for his second infusion. Two weeks following this dose, he was admitted with Grade 3 variceal bleeding (moderate without transfusions needed), and thus underwent urgent EGD with additional banding. He continued to receive a total of four concurrent and adjuvant cycles of atezolizumab without event and remains alive at last follow-up.

Table 4 summarizes the suspected cause of death of the 12 patients who died, with consensus attribution assigned to disease progression, RT toxicity, systemic therapy-related toxicity, tumor-related liver failure (TRLF), RT-related liver failure, comorbidities, or emergent life-threatening episode. TRLF was deemed “definitely” or “possibly” related to demise in four patients. Distant disease progression was attributed as being “definitely” or “possibly” related in five patients. Two patients succumbed to life-threatening emergencies (sepsis and liver tumor hemorrhage). There were no scored instances of RT-related toxicity, RT-related liver failure, or toxicity from atezolizumab plus bevacizumab as being the “possible” or “definite” cause of death.

### 3.4. Autoimmune Toxicity

Two of the 21 patients (9.5%) developed immune checkpoint inhibitor (ICI)-related myocarditis (Grade 3 with cardiac MRI showing left ventricular ejection fraction (LVEF) <50%) [18] or gastroenteritis (Grade 1, <4 bowel movements per day) [19]. There were no rashes. The patient with myocarditis received one course of atezolizumab only 2 weeks prior to his first fraction of RT. The next day, he presented to the ER with new onset sinus tachycardia (heart rate 110 s), with laboratory findings of elevated troponin to 517 ng/mL, but then developed atrial fibrillation with rapid ventricular rate. Cardiac MRI showed LVEF mildly reduced at 45%, with findings suggesting early myocarditis. In the ICU, he received high-dose methylprednisolone (1 g daily for 3 days) and an amiodarone infusion, with reversion to sinus rhythm and down-trending troponin levels. Right and left heart catheterization with endomyocardial biopsy revealed lymphocytic myocarditis. He transitioned to amiodarone by oral administration for 5 weeks, as well as 2 mg/kg oral prednisone with slow taper for 6 weeks. He did not receive any further doses of atezolizumab or bevacizumab but resumed RT to completion without further events. He began lenvatinib 3 weeks after completing RT and remained well with 5 months of follow-up.

The second patient had Grade 1 gastroenteritis and had received seven total infusions of atezolizumab—five neoadjuvant courses of atezolizumab plus bevacizumab, one concurrently with RT (atezolizumab only), and one adjuvant dose of atezolizumab plus bevacizumab following RT completion. Two weeks following completion of RT, when receiving his seventh infusion of atezolizumab plus bevacizumab, the patient reported the recent onset of progressive post-prandial right-sided abdominal pain, cramping, bloating, and 11-pound weight loss spanning 6 months. There was no diarrhea and so his symptoms were initially felt to represent a functional disorder. However, endoscopic biopsies revealed possible immune checkpoint inhibitor-related injury. His eighth cycle of atezolizumab was held, and only bevacizumab was given for two more cycles. The symptoms improved significantly with two intravenous injections of 50 mg hydrocortisone separated by 2 weeks, followed by a methylprednisolone dose pack. He also received three intravenous doses of vedolizumab, an α_4_β_7_ integrin blocker for gut-specific anti-inflammatory activity used for the treatment of ulcerative colitis and Crohn disease, spanning 6 weeks.

### 3.5. Changes in Absolute Lymphocyte Count during RT with Atezolizumab Plus Bevacizumab

**Figure 5:**
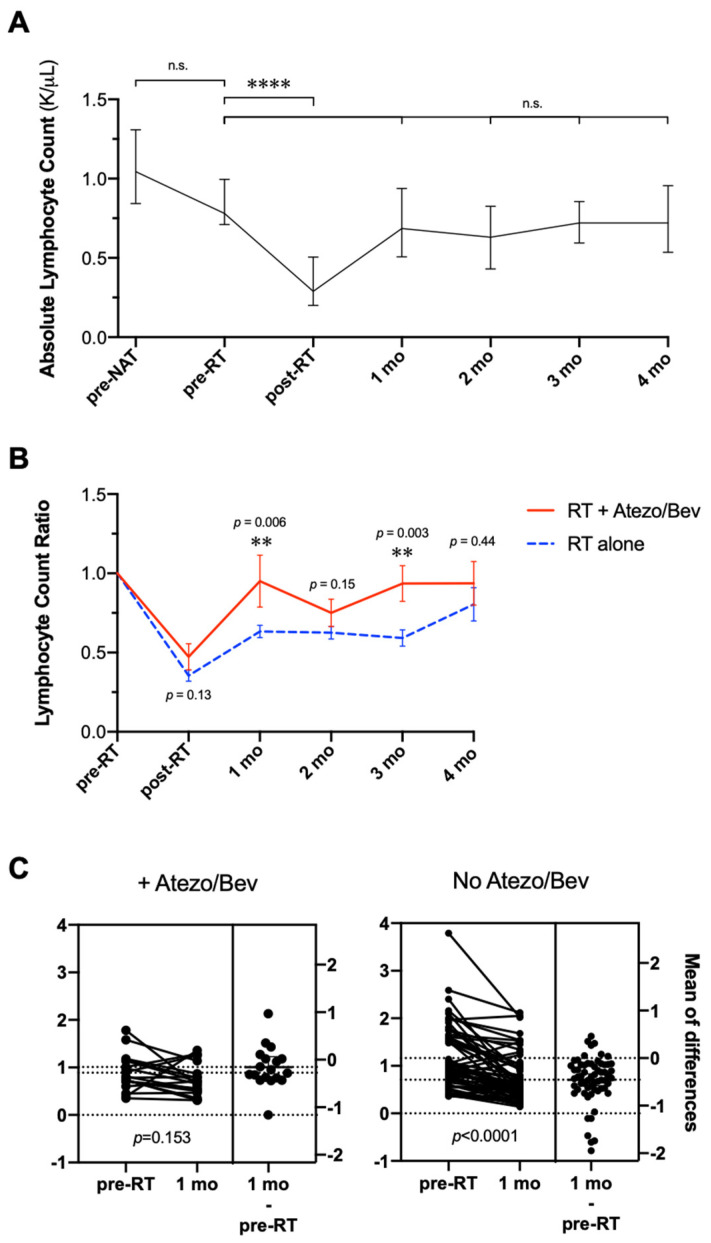
RT with atezolizumab plus bevacizumab is associated with accelerated recovery of lymphopenia. Depicted are absolute lymphocyte counts (**A**) in patients receiving RT with atezolizumab plus bevacizumab in all sequences, shown as median with IQR. (**B**) Post-RT to pre-RT ratios of the ALC up to 4 months post-treatment, comparing patients who received RT with atezolizumab plus bevacizumab in all sequences vs. RT without these therapies (mean ± SEM). (**C**) Individual patient ALC differences 1 month post-RT vs. pre-treatment baseline comparing patients who underwent combined modality therapy vs. RT without. Abbreviations: RT = radiation therapy; ALC = absolute lymphocyte counts; mo = month. *p*-values were derived from parametric Student’s *t* tests. ** *p* < 0.01, **** *p* < 0.0001, n.s. = not statistically significant.

There was a profound decrease in the ALC (Figure 5A) post-RT (*p* < 0.0001). Nadir levels were an average of 47.3% of the pre-treatment baseline. This was restored by 1 month to comparable pre-treatment baseline levels (mean 95.1%), not statistically significant (n.s.). This effect was mostly persistent, with a slight drop in ALC at 2 months (mean 75.1%, n.s.), but restored and remained elevated at 3- and 4 months to the pre-treatment baseline (mean 93.6–93.7%, n.s.).

This cohort was then compared to a previously published cohort from our institution of patients with HCC treated with RT without atezolizumab or bevacizumab [17] in order to examine if the combination of RT and atezolizumab immunotherapy (in the collective sequence) influenced the pace of ALC recovery. Post-RT to pre-RT ratios of the ALC computed at various timepoints (Figure 5B) up to 4 months demonstrated an accelerated return to baseline lymphocyte count among patients who received RT with atezolizumab plus bevacizumab vs. RT without these systemic therapies (*p* < 0.04 at 1 month, n.s. at 2–4 months).

In patients who did not receive atezolizumab or bevacizumab in tandem with RT, the average post-RT/pre-RT ratio was 35.4% of the pre-treatment baseline ALC. The mean ALC ratio rose to 63.3% by 1-month post-RT in these patients (*p* < 0.0001 compared to pre-RT baseline), which was persistently low at 2 and 3 months for an average of 62.5% (*p* < 0.0001) and 59.2% (*p* < 0.0001), before recovery at 4 months to 80.4% (*p* = 0.011 compared to pre-RT baseline).

Individual patient counts 1-month post-RT vs. pre-treatment demonstrated a significantly faster recovery of ALC among patients who received RT with atezolizumab plus bevacizumab (neoadjuvant, concurrent, or adjuvant) vs. RT without these therapies, *p* = 0.006 (Figure 5C).

## 4. Discussion

The majority of patients with HCC have unresectable tumors, recurrence following curative therapy, or are medically unfit for surgery with significant underlying liver disease [20]. Patients often tend to succumb to tumor progression driving liver failure, which is frequently complicated by underlying advanced cirrhosis [13]. Liver directed therapy with external beam RT may be utilized in well-selected patients with HCC, including those with PVTT related to tumor progression, for the purpose of improving local control and quality of life by decreasing morbidity [21]. The judicious use of RT may postpone hilar obstruction from local tumor progression, prevent vascular compromise, and reduce the risk of liver failure [22]. It is conceivable that a combined modality approach may have utility in this disease, where systemic therapy addresses all sites of disease while local radiation therapy decreases the risk of intrahepatic progression causing liver decompensation, vascular compromise, and mortality. Atezolizumab and bevacizumab doublet therapy has emerged as the frontline standard of care for most patients with unresectable or metastatic HCC, replacing targeted systemic therapy such as sorafenib [7]. The safety and efficacy of such combined modality therapy with RT is currently unknown. To our knowledge, this is the first reported experience analyzing outcomes and toxicities of patients with HCC treated with RT in the context of modern systemic therapy with atezolizumab plus bevacizumab. From our findings, the treatment appears to be generally safe, does not potentiate liver decompensation, and confers mostly benign Grade 1 radiation-related side effects of fatigue, nausea, and abdominal pain. Altogether, patients tolerated the treatments well.

This cohort examined in this study consisted of patients with particularly advanced disease and cirrhosis, explaining the poor prognosis of these patients dispositioned to RT and atezolizumab plus bevacizumab. The median OS in this cohort was 16.1 months, compared to 19.2 months in the IMbrave150 study with atezolizumab and bevacizumab without RT [7]. Many factors explain this difference as well as the short OS from the start of RT. In 33.3% of our cohort, the patients were pre-treated with other therapies prior to the initiation of RT, inferring a more advanced disease course. Only 40% of patients in the IMbrave150 trial had macrovascular invasion [7], whereas 71.4% of this cohort treated with RT had this high-risk, poor prognostic feature [13]. Most importantly, 33.3% of the patients in this study had ≥ Child-Pugh B cirrhosis, which had been a criterion for exclusion in the IMbrave150 study and other clinical trials that posited bevacizumab as a potential therapeutic option for unresectable HCC. GI bleeding occurred in three patients (14.3%), but the bleeding was variceal in nature for two of these patients, who notably had Child-Pugh A disease. Among our three cases, GI bleeding was felt to be unrelated to RT due to its occurrence prior to RT in two of the patients or several months after therapy in one patient. Additionally, in this patient, the hemorrhagic episode was temporally disjointed from bevacizumab delivery and thus likely an unfortunate consequence of his progressive disease course. Our reported rate for GI bleeding in this cohort is consistent with historical rates of 10–11% [23,24]. Notably, a Phase II study of concurrent and adjuvant bevacizumab with RT and capecitabine/gemcitabine for locally advanced pancreatic cancer evidenced a small number of bleeding events (6.1%) possibly related to bevacizumab, but this was only in patients whose tumors appeared to be clinically invading the duodenal mucosa. Once patients with duodenal invasion were deemed ineligible for trial enrollment, there were no further hemorrhagic adverse events and thus bevacizumab was felt to be safe to be given with RT [25]. Perhaps the delivery of bevacizumab with RT may be of larger concern within the scope of HCC, due to the incidence of portal venous pathophysiology and esophageal varices. Ultimately, independent of RT, the retrospective evaluation of our experience suggests that oncologists are utilizing clinical discretion to decide which patients with ≥ Child-Pugh B cirrhosis may still tolerate therapy with atezolizumab plus bevacizumab. Additionally, GI bleeding is a possible adverse outcome even in those with little to no signs of liver decompensation with Child-Pugh A disease. To comprehensively portray the risk of this therapy in a manner that is faithful to real-world dispositions, prospective studies may consider including well-selected patients with Child-Pugh B disease and negative endoscopic examinations with a plan for a priori stratification analyses. Furthermore, to underscore an important point, we prevented RT-induced GI bleeding by conforming to strict planning considerations as outlined in our methods. For hypofractionated schedules (10–15 fractions), we ensure that the maximum dose to the stomach and duodenum is less than 45 Gy.

ICI-related autoimmune adverse events occurred in 9.5% of the patients, with the development of gastroenteritis and myocarditis in two patients. Rates of ICI-colitis from atezolizumab plus bevacizumab have not been previously reported. However, any grade of diarrhea with this therapy combination were reported to have occurred in 18.8% of patients, and Grade 3 or 4 diarrhea happened in 1.8% of the patients [26]. Historical rates of myocarditis have also not been reported. There is more information on autoimmune adverse events with other ICIs, which beckons closer study of the incidence of such side effects in patients receiving atezolizumab plus bevacizumab.

We were intrigued by the significantly faster recovery of ALC among patients who received RT with atezolizumab plus bevacizumab vs. RT without these therapies. The correlation of treatment-related lymphopenia with adverse outcomes in patients has been widely documented in a broad range of malignancies. High-grade lymphopenia was associated with increased risk of death in a combined cohort of solid tumors across all pathologies, hazard ratio (HR) of 2.1, 95% CI: 1.54–2.78; *p* < 0.0001 [27]. High-grade lymphopenia in patients with HCC undergoing RT had worse OS (13.6 vs. 46.7 months, *p* < 0.001) [28]. We also have previously shown that lymphopenia associated with RT may portend a poor prognosis in HCC, with G3 or higher lymphopenia acting as an independent predictor of worse survival [17]. This would suggest that hastened recovery of ALC with atezolizumab is a favorable prognosticator. As a multi-targeted immunomodulator with anti-PD-L1 and B7 (CD80) costimulatory binding to reverse T cell exhaustion [29], it can be extrapolated that atezolizumab offers enhanced lymphopoiesis and count recovery, with mitigation of prolonged lymphopenia from RT. We note limitations in this analysis, including the small size of this cohort and heterogeneity of the patients. Ultimately, confirmation of this mechanistically as well as in prospective studies may be of interest. It would be curious to investigate if such impacts on ALC are also seen in patients undergoing therapy with single-agent nivolumab (CheckMate 459 and CheckMate 040), nivolumab plus ipilimumab (CheckMate 040), lenvatinib plus pembrolizumab (LEAP-002), doublet tremelim plus tremelimumab (HIMALAYA), or cabozantinib plus atezolizumab (COSMIC-312), which have all been investigated for advanced HCC [30,31,32,33,34,35].

We previously described that proton therapy may mitigate lymphopenia compared with photon therapy, potentially due to reduced dose exposure of sites of lymphopoiesis, with less “low-dose bath”, and a lower spleen dose [17]. However, it is unlikely that the differences we observed in ALC count with atezolizumab plus bevacizumab could be attributed to differences in the proportion of patients treated with proton therapy. In the comparative analysis of the previous dataset without systemic therapy vs. the current set of patients who received RT with atezolizumab plus bevacizumab, 28.2% vs. 33.3% of the patients received proton therapy, respectively, indicating comparability of the cohorts. With a larger sample size, an additional subset analysis to elucidate the differential impact of atezolizumab plus bevacizumab in modulating treatment-related lymphopenia from proton therapy vs. IMRT may be enlightening, since the mitigation in ALC drop with proton therapy has been correlated with superior prognosis.

Outside of the small number of patients and limited follow-up, other limitations that should be noted include this being a retrospective study subject to selection biases that may confound interpretation of the efficacy of these treatments together. The patients in this study were highly heterogeneous in their radiation regimens, disease stage, degree of liver dysfunction, and sequencing of atezolizumab and bevacizumab in relation to RT, which may dilute specific conclusions. Longer follow-up would better elucidate the radiographic outcomes of irradiated lesions, where local control of the irradiated tumors was noted to be excellent. However, in most patients receiving systemic therapy for HCC, local control is not the main driver of mortality. Additionally, a third of the patients in this study received other therapies before undergoing treatment with atezolizumab plus bevacizumab. This may actually be more faithful to clinical practice, where paradigm shifts in management may be translated to heavily pretreated patients as salvage therapies as opposed to intended frontline incorporation. Ultimately, the high-risk features of this cohort help explain the poor survival in this cohort since both RT and atezolizumab plus bevacizumab may have been introduced later in the disease course in the context of more advanced burden of cancer, with more advanced Child-Pugh B cirrhosis and the majority of the patients having PVTT.

From our findings, practitioners can feel more confident that RT is an acceptable concurrent or immediate adjuvant modality in patients receiving atezolizumab plus bevacizumab. In fact, this approach may be advantageous if the hastened pace of recovery of lymphopenia is extrapolated to indicate a more favorable disease course. Confirmation of this suggestion in future prospective studies as a biomarker for prognostic outcome may be particularly provocative. Prior to the landmark adoption of atezolizumab plus bevacizumab as the new standard of care for HCC, the Phase III trial RTOG 1112 was examining the efficacy and safety of sorafenib ± SBRT for patients with HCC. These results would have supplied evidence regarding the utility and indications for combined modality therapy for this disease. However, with the trial now closed and overhaul of sorafenib by atezolizumab plus bevacizumab as the new standard of care, results from RTOG 1112 may be challenging to interpret. Currently, the ongoing single-center, single-arm pilot study NCT04857684 is designed to evaluate the safety and tolerability of neoadjuvant RT plus atezolizumab and bevacizumab in patients with treatment-naïve resectable HCC, with secondary outcomes including response rates, surgical success, and survival. This will further provide clarity regarding the value of radiation therapy in the management of HCC in the context of modern doublet immunotherapy-antiangiogenic therapy combinations.

## 5. Conclusions

Liver directed RT may be utilized in well-selected patients with HCC, including those with PVTT related to tumor progression, for the purpose of improving local control and decreasing morbidity by avoiding central liver obstructive complications. With the adoption of atezolizumab plus bevacizumab as the frontline standard of care for the treatment of patients with unresectable or metastatic HCC, it is important to assess the safety and outcomes of combined modality therapy with liver-directed RT. In this first reported experience of HCC patients treated with RT in the context of modern systemic therapy with atezolizumab plus bevacizumab, combination therapy does not potentiate liver decompensation, and it appears to be safe and well-tolerated. As a favorable prognosticator, there appears to be a significantly faster recovery of ALC among patients who received RT with atezolizumab plus bevacizumab versus RT without this systemic therapy combination. Confirmation of this suggestion as a biomarker for prognostic outcome in a randomized clinical trial may be insightful.

## Figures and Tables

**Table 1:** Patient, tumor, and treatment characteristics.

Patient Characteristics		*n*	%
**Total No.**		21	
**Age**			
	Median (range), years	68 (38–78)	
**Sex**			
	Male	17	81%
**Child–Pugh Score (CPS)**, pre-RT			
	5 A	10	47.6%
	6 A	4	19%
	7 B	2	9.5%
	8 B	2	9.5%
	9 B	2	9.5%
	10 C	1	4.8%
**BCLC**			
	A	1	4.8%
	B	2	9.5%
	C	17	80.9%
	D	1	4.8%
**Clinical stage at diagnosis**			
	rpIB	1	4.8%
	II	1	4.8%
	IIIA	3	14.3%
	IIIB	7	33.3%
	IVA	3	14.3%
	IVB	6	28.6%
**PV Tumor Thrombus (PVTT)**			
	Yes	15	71.4%
	No	6	28.6%
**Etiology**			
	Viral (HBV or HCV)	14	66.6%
	Metabolic syndrome	6	28.6%
	Alcoholic cirrhosis	1	4.8%
**T-stage**			
	rpT1 b, T2, T3	6	28.6%
	T4	15	71.4%
	Median GTV (range), cc^3^	149.4 (25.1–3426.9)	
	Median irradiated tumor diameter (range), cm	9.1 (3.9–18.6)	
	Median of “mean liver–GTV” dose (IQR), Gy	17.4 (12.2–19.9, max 21.9)	
	Volume of liver receiving ≤20 Gy (IQR), cc^3^	331.2 (175.6–509.3, max 628.7)	
**N-stage**			
	N0	15	71.4%
	N1	6	28.6%
**M-stage**			
	M0	15	71.4%
	M1	6	28.6%
**Modality**			
	IMRT	13	61.9%
	Proton	7	33.3%
	3 D	1	4.8%
**Treatment**			
	Median total dose (range), Gy	60 (20–75)	
	Fractions (range)	15 (5–25)	
**BED_10_ (Gy)**			
	>70 Gy (mean 89.3 Gy)	17	81%
	28–56 Gy	4	19%

Abbreviations: RT = radiation therapy; cc^3^ = cubic centimeters; GTV = gross tumor volume; IMRT = intensity modulated radiation therapy; BED_10_ = biologically effective dose calculated at an α/β ratio of 10.

**Table 2:** Timing and use of prior therapies targeting HCC in the seven patients with prior treatment.

Prior Therapy (Interval between First Dose of Prior Therapy and Initiation of A/B, Months)
**TACE** with doxorubicin (6, 3)
**Lenvatinib** (4), one dose with poor tolerance
**Nivolumab** (31), lost to follow-up until reemergence
**Lenvatinib** (3)
**Lenvatinib** (7), **Nivolumab** (4), **Ramucirumab** (1)
**Resection** (50), **RFA** (34), **sorafenib** (21), **nivolumab** (17), **regorafenib** (14)
**Lenvatinib** (23), **Nivolumab** (16)

**Table 3:** Provider-scored acute toxicities of RT with atezolizumab plus bevacizumab.

	Pain	Fatigue	Nausea	Vomiting	Diarrhea	Constipation	Dermatitis
Grade 1	7/21 (33.3%)	12/21 (57.1%)	10/21 (47.6%)	2/21 (9.5%)	4/21 (19.1%)	2/21 (9.5%)	3/21 (14.3%)
Grade 2	1/21 (4.8%)	1/21 (4.8%)	1/21 (4.8%)	1/21 (4.8%)	0/21 (0%)	0/21 (0%)	0/21 (0%)

**Table 4:** Cause of death with attribution to suspected etiology.

Patient	1	2	3	4	5	6	7	8	9	10	11	12
Distant POD	◊◊◊◊	⋅	⋅	◊	⋅	◊◊◊	◊◊◊◊	◊◊◊	⋅	◊	⋅	◊◊◊◊
RT toxicity	⋅	⋅	⋅	⋅	◊	⋅	⋅	⋅	⋅	⋅	⋅	⋅
A/B toxicity	⋅	⋅	⋅	⋅	◊	⋅	⋅	⋅	⋅	⋅	⋅	⋅
TRLF	⋅	⋅	⋅	◊◊◊◊	⋅	⋅	⋅	⋅	◊◊◊	◊◊◊	◊◊◊	⋅
RT-related LF	⋅	⋅	⋅	⋅	⋅	⋅	⋅	⋅	⋅	⋅	⋅	⋅
Comorbidity	⋅	◊◊◊	◊◊◊◊	⋅	⋅	◊	⋅	⋅	⋅	⋅	⋅	◊
Emergent	⋅	⋅	◊◊◊◊	⋅	◊◊◊◊	⋅	⋅	⋅	⋅	⋅	⋅	⋅

Consensus scale used was ⋅ (not related), ◊ (unlikely related), ◊◊◊ (possibly related), and ◊◊◊◊ (definitely related). Abbreviations: POD = progression of disease; RT = radiation therapy; A/B = atezolizumab plus bevacizumab; TRLF = tumor-related liver failure; LF = liver failure.

## Data Availability

Not applicable.
